# Soil Moisture Estimation by Assimilating L-Band Microwave Brightness Temperature with Geostatistics and Observation Localization

**DOI:** 10.1371/journal.pone.0116435

**Published:** 2015-01-30

**Authors:** Xujun Han, Xin Li, Riccardo Rigon, Rui Jin, Stefano Endrizzi

**Affiliations:** 1 Cold and Arid Regions Environmental and Engineering Research Institute, Chinese Academy of Sciences, Lanzhou, PR China; 2 CAS Center for Excellence in Tibetan Plateau Earth Sciences, Chinese Academy of Sciences, Beijing, PR China; 3 Department of Civil and Environmental Engineering, University of Trento, Trento, Italy; 4 Department of Geography, University of Zurich, Zurich, Switzerland

## Abstract

The observation could be used to reduce the model uncertainties with data assimilation. If the observation cannot cover the whole model area due to spatial availability or instrument ability, how to do data assimilation at locations not covered by observation? Two commonly used strategies were firstly described: One is covariance localization (CL); the other is observation localization (OL). Compared with CL, OL is easy to parallelize and more efficient for large-scale analysis. This paper evaluated OL in soil moisture profile characterizations, in which the geostatistical semivariogram was used to fit the spatial correlated characteristics of synthetic L-Band microwave brightness temperature measurement. The fitted semivariogram model and the local ensemble transform Kalman filter algorithm are combined together to weight and assimilate the observations within a local region surrounding the grid cell of land surface model to be analyzed. Six scenarios were compared: 1_Obs with one nearest observation assimilated, 5_Obs with no more than five nearest local observations assimilated, and 9_Obs with no more than nine nearest local observations assimilated. The scenarios with no more than 16, 25, and 36 local observations were also compared. From the results we can conclude that more local observations involved in assimilation will improve estimations with an upper bound of 9 observations in this case. This study demonstrates the potentials of geostatistical correlation representation in OL to improve data assimilation of catchment scale soil moisture using synthetic L-band microwave brightness temperature, which cannot cover the study area fully in space due to vegetation effects.

## Introduction

Soil moisture plays an important role in the catchment scale water cycle and land-atmosphere interactions [1,2,3]. The satellite missions of soil moisture provide us the opportunity to measure the large scale land surface soil moisture from space [4,5]. The land surface / hydrologic models also become the important tools for the soil moisture profile estimation at the global, regional and catchment scale [1,6,7,8,9]. In order to improve the performance of the model simulation, the studies of land data assimilation have made rapid progress to integrate the numerical model estimations of land surface states and the observations from remote sensing and ground based instrument to improve the characterizations of the water and energy cycle [10,11,12,13,14]. In the land data assimilation, it is very common that all model grid cells cannot be measured at the same time due to the spatial availability of the measurements (e.g., the limited coverage of microwave sensors because of dense vegetation, limited coverage of thermal sensors because of cloud or the limited measurement scale of ground based sensors) [15,16,17]. Thus, the question of how to carry out the data assimilation for the model grid cells lack of observations has been proposed, and studies have paid more attention to the spatial horizontal transfer of observations in the data assimilation, in which the model states could be updated using the local correlated observations [18,19,20].

Two main strategies can be undertaken to utilize the local correlated observations through the horizontal spatial correlation characteristics of land surface variables in data assimilation [21,22,23]: (1) use the correlated information contained in the model forecast covariance, in which the spatial horizontal correlations among different model locations can be described with the covariance; and (2) use the observational correlation information where the spatial horizontal correlations are defined through the correlated observations. The first method is often applied with the ensemble Kalman filter (EnKF), which has been studied in numerous land data assimilation applications because of its conceptual formulation and relative easy implementation [18,20,24], but the inverse operation, storage of matrices and parallel computing for the large scale application in the first method with 3D-EnKF are difficult [25]. So the second approach with local ensemble transform Kalman filter (LETKF) becomes more and more popular because of its efficient parallel implementation in technique [26,27].

Both EnKF and LETKF use the ensemble representation of the background error covariance. Due to the computational limits, small ensemble members (compared with the degree of freedom of the system) are usually used in calculations. This could result in large sampling errors in the approximation of background error covariance [28,29] and produce spurious large magnitude correlations among the long-range separated model grid cells [18,30]. The spurious large magnitude correlations will assign a large weight to the far away observations, and is contrary to the reality.

In order to reduce the impacts of spurious long-range correlation on the assimilation performance, the covariance localization (CL) techniques are first proposed in the estimation of the background error covariance of EnKF. With CL, one can allow observations having great influences on the adjacent model grid cells and small influences on the far model grid cells. The so-called Schur product [18,29,31] is used in the CL to multiply the ensemble approximation of the background error covariance matrix with a distance-dependent correlation function to suppress the distant correlations. This localization limits the impacts of distant observations. On the other hand, observation localization (OL) has also been proposed for LETKF in atmospheric data assimilation recently and is often used to filter out the small correlations associated with the distant observations [29,31]. In OL, the observation error covariance matrix is divided by a distance-dependent correlation function to increase the observation error variance of distant observations and to reduce their weights in data assimilation [27,29,30,31]. For each model grid cell, local correlated observations need to be selected and used in OL to do the analysis.

Both CL and OL have been proved to perform similarly in the data assimilation [29,31]. However, CL needs to calculate the whole background covariance matrix for all model grid cells and will result in large memory requirement for large number of model grid cells, and need careful parallelization implementation [25]. This is not trivial in practice. With OL, the large amount model grid cells can be partitioned into small blocks to avoid the memory limits, and the assimilation for each model grid cell of these blocks can be separated and parallelized efficiently. Thus, we wanted to evaluate OL in land data assimilation by assimilating the synthetic L-band microwave brightness temperature data into the Community Land Model (CLM) [32] to improve the soil moisture profile characterization. We did this by taking into account the spatial correlations and vegetation influence [15] of microwave brightness temperature data to compensate the shortcomings of remote sensing in spatial coverage.

The OL scheme can limit the effect of faraway observations and filters out the small correlations associated with these observations, and it has been extensively applied in the LETKF [19,27,31] and other EnKF variants [29] to assimilate the local surrounded observations by taking the spatial correlation into account. In LETKF, we can perform the data assimilation grid cell by grid cell in parallel, and only the limited number of local surrounded observations will be assimilated for each grid cell. The selection criteria of the local observations to be assimilated depends on the spatial correlation characteristics of the observation data. Thus, a distance-dependent correlation function should to be defined to describe the spatial correlation characteristics represented by the observation data.

The local correlated observations need to be chosen for each model grid cell based on the distance-dependent correlation function and spatial correlation characteristics before the data assimilation in OL. This step, however, is very subjective. Moreover, the subjective distance-dependent function and constant correlation range were used in the localization [31,33,34]. A commonly used function is called Gaspari and Cohn model in the atmospheric assimilation field [18].

There are no general rules to define the distance-dependent spatial correlation function for both CL and OL. In this study we try to use the geostatistical methods [23] to practically get the more reasonable representation of this subjective distance-dependent correlation function. This function determines which observations could be used in the assimilation for each model grid cell based on the correlation characteristics of the observation data. In particular, we use the geostatistical semivariogram model to describe the correlation among observations [35,36]. Geostatistical semivariogram models have been successfully used in the analysis of soil moisture spatial pattern [23,37,38].

The objective of this study is whether we can use the small coverage observations to update the whole study area, even only 1 local microwave brightness temperature is assimilated for each model grid cell. And whether the geostatistical semivariogram analysis can be used to characterize the observational spatial correlation. Here, we evaluated this assumption in a observing system simulation experiment, where we additionally used the distributed land surface CLM [32] to simulate the soil moisture evolution at the catchment scale. In CLM, the synthetic L-band microwave brightness temperature observation derived from a microwave radiative transfer model were assimilated by LETKF and OL, and the impacts of the forest on the microwave brightness temperature observation was also considered by reducing the spatial coverage of the synthetic observation. Six scenarios were carried out to evaluate the impacts of assimilating different number of local correlated observations in OL on the assimilation results, while considering the horizontal spatial correlations of the microwave brightness temperature and the spatial availability of observations.

The paper is organized as follows. In section 2, we describe the LETKF. In section 3, we briefly outline the geostatistics theory required to analyze the necessary horizontal spatial correlation of observation. Section 4 introduces the preparation of the synthetic assimilation experiment. Section 5 evaluates the proposed method in a synthetic microwave brightness temperature assimilation experiment. Section 6 presents the summary and discussion.

## Materials and Methods

### 1. Local Ensemble Transform Kalman Filter

LETKF [27] uses the OL approach to do the local analysis in the framework of the square root variant of the classical EnKF [39], in which the observations from a local region surrounding the model grid cell to be analyzed are first selected. Then the OL is done by increasing the observational error covariance of the observations that are far away from the model grid cell, and observational error covariance of chosen observation is divided by a distance-dependent correlation function. The OL suppresses the impacts of the long-range observations on the analysis [29,40]. In the OL analysis scheme, the model grid cell is assimilated separately. For each model grid cell, the corresponding local observations will be chosen based on the observational spatial correlation characteristics. It should be noted that some of the observations used for a particular model grid cell will also be used in the analysis of other neighboring grid cells. This imposes a smoothing effect from one grid cell to its neighbors. In practice, a distance-dependent correlation function (by fitting of the two-point correlation matrix to a distance-dependent correlation function using geostatistics) and its correlation range need to be defined, and the correlation range defines the threshold above which the data assimilation analysis ignores the correlation. The LETKF code used is adapted from Google website (Available: http://code.google.com/p/miyoshi. Accessed 2014 Dec 11). The details of LETKF computation can be found in Hunt et al. (2007).

There are two analysis steps in LETKF. One is the global analysis, in which two global matrixes are constructed using the model forecast ensemble members:

$$X^{b}=\left[x_{1}^{b}-{\bar{x}}^{b},\ldots ,x_{N}^{b}-{\bar{x}}^{b}\right]$$ (1)

$$y_{i}^{b}=H\left(x_{i}^{b}\right)$$ (2)

$$Y^{b}=\left[y_{1}^{b}-{\bar{y}}^{b},\ldots ,y_{N}^{b}-{\bar{y}}^{b}\right]$$ (3)

where $x_{1}^{b},\ldots x_{N}^{b}$ are the model forecast ensemble members, *N* is the ensemble size, ${\bar{x}}^{b}$ is the ensemble mean of $x_{1}^{b},\ldots x_{N}^{b}$, and *H* is the observation operator (it is CMEM model in this study). The expression ${\bar{x}}^{b}$ is composed of one weighted soil moisture (explained in section IV) and 10 layers of soil moisture in soil moisture assimilation. So the dimension of ${\bar{x}}^{b}$ is 11.

The other is called local analysis in which the selected local observations for each model grid cell are used to calculate the local analysis error covariance and the perturbations in the ensemble space:

$${\text{P}}^{a}=\left[\left(N-1\right)I+Y^{bT}R^{-1}Y^{b}\right]$$ (4)

$${\text{W}}^{a}={\left[\left(N-1\right){\text{P}}^{a}\right]}^{1/2}$$ (5)

$${\bar{\text{w}}}^{a}={\text{P}}^{a}Y^{bT}R^{-1}\left(y^{o}-{\bar{y}}^{b}\right)$$ (6)

$$X^{a}=X^{b}{\text{W}}^{a}+{\bar{x}}^{b}$$ (7)

where *R* is observation error covariance, *y^o^* is the observation vector, and *X^a^* is the analysis model ensemble members.

### 2. Correlation Functions and Geostatistics

The horizontal spatial correlation of the soil moisture among adjacent grid cells has been reported in the soil moisture spatial variability studies, and the distance of isotropic horizontal spatial correlation ranges from tens of meters to tens of kilometers [21,22,23]. However, the horizontal spatial correlation of soil moisture changes over time as the environment condition changes. Precipitation and land surface features, such as the soil texture and land cover, determine the scales of the horizontal spatial correlation structure. For example, soil moisture tends to be highly horizontally correlated after a prolonged wet period. During the dry down period, the horizontal correlations tend to decrease. However, after a long period of dry down, the entire basin becomes dry, and there will be strong horizontal spatial correlation [23,41]. Because of the strong relationship between soil moisture and microwave brightness temperature, there should be similar spatial correlation pattern in the brightness temperature data [42].

Geostatistics provides both the theory and tools, with which we are able to describe the horizontal spatial correlation patterns contained in the observation data. Several geostatistical semivariogram models could be used to fit the patterns of horizontal spatial correlations of the microwave brightness temperature. The distance-dependent correlation function of OL for soil moisture could be inferred from the semivariogram *γ(h)* analysis related to the two point covariances. We assumed that the semivariogram is isotropic in our experiment for simplicity. The semivariogram models we choose were the Gaussian model, the exponential model, the spherical model, and the Matern model [36,43]:

$$\gamma (h)=c_{0}+c(h/a*(1.5-0.5*{\left(h/a\right)}^{2}))$$ (8) spherical

$$\gamma (h)=c_{0}+c(1-exp(-3h/a))$$ (9) exponential

$$\gamma (h)=c_{0}+c(1-exp(-(3h^{2}/a^{2})))$$ (10) Gaussian

$$\gamma (h)=c_{0}+c(\frac{1}{2^{v-1}\Gamma (v)}{\left(\frac{2v^{1/2}h}{a}\right)}^{v}K_{v}(\frac{2v^{1/2}h}{a}))$$ (11) Matern

where *c*_0_ is the nugget, *c* is equal to the sill minus nugget, *h* is the distance among grid cells, *a* is the (effective) correlation rang, *K_v_* is a modified Bessel function of second order *v*, Γ is the gamma function, and *v* (kappa) is called “smoothness parameter” (*v* > 0). The normalized semivariogram *γ*(*h*)^*Nor*^ is defined as *γ*(*h*)^*Nor*^ = *γ*(*h*)/(*c*_0_ + *c*). The correlogram is then derived by 1 − *γ*(*h*)^*Nor*^.

The final correlogram value will be normalized to the range of [0∼1] by the maximum correlogram value, which means that the correlogram value at the observation location is equal to one. It will decrease towards to zero as the distance from the model grid cell increases gradually, and will become null when the spatial distance is greater than the specified correlation range. The scheme of OL in LETKF is as follows: for each model grid cell, the spatial correlation of the microwave brightness temperature data will be modeled using the best fitted semivariogram model at each assimilationg step. The microwave brightness temperature data whose correlograms are greater than a predefined threshold (we used 0.1 in this paper) will be chosen and used in the subsequent assimilation of each model grid cell.

### 3. Study Area, Models, and Experiment Setup

There are no specific permissions required for these locations/activities and the field studies did not involve endangered or protected species.

#### 3.1. Study Area

**Figure 1:**
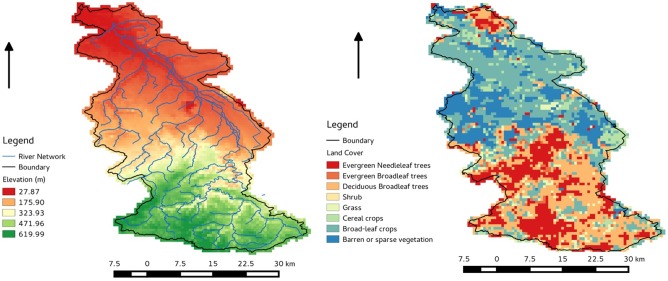
The elevation (left) and MODIS plant functional type (right) of Rur Catchment.

The study is the Rur Catchment (51°10′13″N–50°22′38″N; 5°55′50″E–7°5′42″E), which is near the Belgian-Dutch-German border, with an area of 2354 km^2^. The annual precipitation is 850–1300 mm/year, and the annual potential evapotranspiration of the southern area is 450–550 mm/year, whereas the corresponding values for the northern area are 650–850 mm/year and 580–600 mm/year, respectively. Forest and grassland dominate the south, whereas fertile agricultural land predominates in the north [44]. The Terrestrial Environmental Observatories (TERENO) initiative [45] has selected Rur for the long-term land surface measurement. Additionally, the Rur Catchment is a validation site for the Soil Moisture and Ocean Salinity (SMOS) mission of European Space Agency (ESA) [5] and Soil Moisture Active-Passive (SMAP) mission of National Aeronautics and Space Administration (NASA) [4] with an extensive wireless sensor network [46] and microwave sensors for soil moisture observation [47]. Fig. 1 shows the elevation map and the plant functional type from MODIS for the Rur catchment.

#### 3.2. Land Surface Modeling

The land surface model CLM (Version 4.5) [32] was chosen as the model operator to simulate the catchment scale soil moisture, soil temperature, and surface fluxes dynamics. The different land surface processes of land biogeophysics, hydrologic cycle, biogeochemistry, human dimensions, and ecosystem dynamics, and land surface heterogeneity are considered in CLM [32]. The geographic longitude-latitude projection was used to prepare the input data for CLM in the spatial resolution of 0.008333 degree (approximately 750 m). The Rur Catchment contains 4340 active model grid cells. The MODIS 500 m product MCD12Q1 of plant functional type [48] was resampled to 0.008333 degree using the nearest neighbor method and was used to define the plant functional type of CLM. The Harmonized World Soil Database v1.2 (HWSD) was used to prepare the soil texture and organic matter density of CLM [49]. The two layers of soil data in HWSD were linearly interpolated to 10 layers for CLM. The forcing data of Global Land Data Assimilation System (GLDAS) atmospheric [50] were interpolated into the model resolution using the MicroMet model [51]. The MODIS leaf area index product (MCD15A3) was used as the monthly leaf area index of CLM. The details of input preparation can be found in [19].

#### 3.3. Community Microwave Emission Model

The sensitivity of the passive microwave brightness temperature to the soil moisture is very significant in L-band frequency and H polarization [42]. The new flexible Community Microwave Emission Modeling (CMEM) Platform was used as the forward model to map the L-band brightness temperature from the soil moisture of CLM [52,53]. CMEM is one of the core components in the soil moisture retrieval algorithm of the SMOS mission [5]. Moreover, CMEM is the result of an extensive review of the current knowledge for the microwave emission and could simulate the brightness temperature of various land cover types.

The top of atmosphere brightness temperature *TB_toa, p_* for polarization *p* can be written as

$$TB_{\mathrm{toa},p}=TB_{\mathrm{au},p}+\exp(-\tau_{\mathrm{atm},p})TB_{\mathrm{tov},p}$$ (12)

$$TB_{\mathrm{tov},p}=TB_{\mathrm{soil},p}\exp(-\tau_{\mathrm{soil},p})+TB_{\mathrm{veg},p}(1+\exp(-\tau_{\mathrm{veg},p}))+TB_{\mathrm{ad},p}\gamma_{r,p}\exp(-2\tau_{\mathrm{veg},p})$$ (13)

where *TB_au, p_* (K) is the up-welling atmospheric emission, and *τ_atm, p_* is the atmospheric optical depth. The expression *TB_tov, p_* (K) is the top of vegetation brightness temperature when the vegetation is represented as a single scattering layer above rough surface; *TB*_soil, *p*_ (K), *TB*_veg, *p*_, and *TB*_ad, *p*_ (K) are the soil, vegetation layer, and downward atmospheric contributions, respectively; *γ_r,p_* is the soil reflectivity of the rough surface; *τ_veg, p_* is the vegetation optical depth along the viewing path.

CMEM uses four modules to compute the contributions from soil, vegetation, snow and atmosphere. The soil module of CMEM contains four components to compute the soil dielectric constant, the effective temperature, the smooth soil emissivity, and the rough soil emissivity. The soil emissivity model describes the relationship between the soil emissivity and the soil dielectric constant, in which the Fresnel equation or Wilheit model [54] can be used. The Wilheit model is a more physically based model and accounts for both the coherent and the incoherent components of signal. The Wilheit model describes the soil as a stratified medium where the soil dielectric constant and the soil temperature vertical profiles are used to compute the air-soil interface emission. The soil moisture of different layers are weighted in the Wilheit model to obtain the weighted soil moisture. This weighted soil moisture can be regarded as the measured soil moisture from the view of microwave sensor.

The input data of CMEM include soil moisture, soil temperature, leaf area index, vegetation type, soil texture and air temperature. The first seven layers of soil moisture and soil temperature of CLM were used in CMEM, which also used the same soil texture, air temperature, leaf area index and vegetation type as CLM. The default parameterizations of CMEM were used in this experiment except for the Wilheit model [54]. This model was used for the smooth surface emissivity, because it considers the signal contributions of different soil layers to the microwave brightness temperature.

#### 3.4. Ensemble Generation

The uncertain model input parameters and atmospheric forcing data are usually used to describe the uncertainties contained in the land surface model. The main sources of model uncertainties in the land surface model include the inaccurate vegetation/soil ancillary parameters, inaccurate model physics, errors in the forcing data and initial conditions. In this data assimilation application, only the random perturbations of uncertain forcing data and soil parameters were provided for CLM to generate 20 random ensemble replicates of model simulations. The spatial-temporal correlated noises using the fast Fourier transform [55] were applied in precipitation, shortwave radiation, longwave radiation and air temperature, in which the physically consistent perturbations (such as a positive perturbation of the shortwave radiation, a negative perturbation of the longwave radiation, and a positive perturbation of air temperature) were generated to conserve the atmospheric balance between radiation, clouds and air temperature [56]. Table 1 summarizes the perturbation parameters used in many studies [57,58]. The temporal correlation was imposed by a first-order auto-regressive model [56,59].

**Table 1:** Summary of perturbation parameters for atmospheric forcing data.

**Variables**	**Noise**	**Standard Deviation**	**Time Correlation Scale**	**Spatial Correlation Scale**	**Forcing Cross Correlation**
Precipitation	Multiplicative	0.5	24 h	10 km	[ 1.0, −0.8, 0.5, 0.0,
Shortwave radiation	Multiplicative	0.3	24 h	10 km	−0.8, 1.0, −0.5, 0.4,
Longwave radiation	Additive	20 W/m^2^	24 h	10 km	0.5, −0.5, 1.0, 0.4,
Air temperature	Additive	1 K	24 h	10 km	0.0, 0.4, 0.4, 1.0]

The soil sand fraction, soil clay fraction, and soil organic matter density were used in CLM to derive the hydrologic and thermal parameters. Thus, we perturbed the sand fraction, clay fraction and organic matter density with uniform distributed noise in the range of [-10%,10%].

#### 3.5. Experiment Setup

In order to evaluate the assimilation results at the catchment scale, an observing system simulation experiment was proposed. A reference run of CLM (single CLM) from 1 April. 2010 to 30 June 2010 was used as the truth for comparison with true sand fraction, true clay fraction, and true organic matter density. One-year spin up was used for the reference run and open loop run before the data assimilation period. The 20 ensemble members of CLM were driven from 1 April 2010 to 30 June 2010 with different perturbed forcing data and soil parameters, and were used as the open loop run for comparison. The true sand fraction was multiplied by 0.5, the true clay fraction was multiplied by 1.5, and the true organic matter density was multiplied by 2.0 to impose the model bias on CLM. The same perturbed soil inputs were used in both the open loop run and data assimilation.

The synthetic L-band (1.4 GHz) brightness temperature data were prepared through the CMEM model using the soil moisture and the soil temperature data from the reference run. The spatially correlated noises were then imposed on the synthetic brightness temperature data using the geostatistical stochastic simulation approach [36], in which the spatially correlated Gaussian random field with mean 0.0 K and an exponential semivariogram model with nugget 0.0 K^2^, variance 4.0 K^2^, and range 10 km were simulated for each data. The brightness temperature data at 06:00Z every three days were collected as the synthetic observation data. There were 31 brightness temperature observations in the whole assimilation time series.

**Figure 2:**
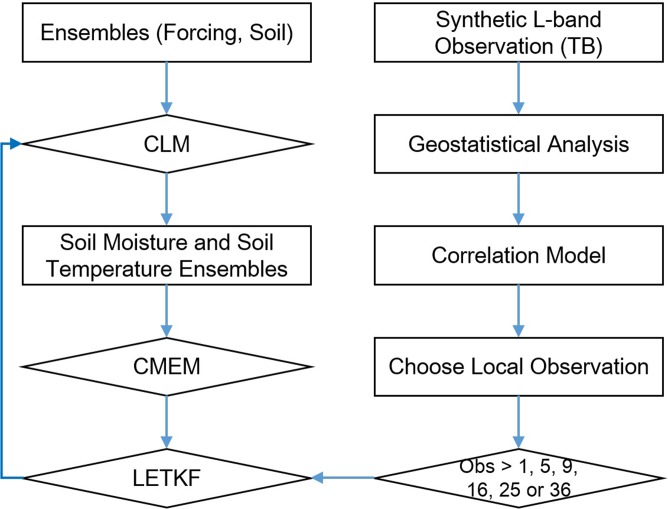
The data assimilation flow chart.

The experiment flow can be summarized in Fig. 2.

### 4. Brightness Temperature Data Assimilation

#### 4.1. Quality Control and Spatial Correlation

In dense or high-vegetation regions, soil moisture cannot be accurately retrieved from the passive microwave brightness temperature [15]. Therefore, the synthetic brightness temperature observation located at the forest regions, where the soil moisture information is difficult to retrieve, were dropped in this study [60]. Then the actual amount of observations that could be assimilated was decreased. Thus, the catchment grid cells could not be covered simultaneously (i.e. only the non-forest area contains the observation data). The Rur catchment was discretized into 4340 grid cells with 2645 grid cells with valid observations. Fig. 1 clearly shows forest distribution which will be used to mask the synthetic microwave brightness temperature observation. To improve the estimation of whole catchment, the observation data should be propagated to the uncovered regions from the covered regions by use of the horizontal spatial correlation among the observations with OL.

In order to represent the spatial correlation characteristics contained in the L-band brightness temperature data, we used the R (R website. Available: http://www.r-project.org. Accessed 2014 Dec 11) geostatistic package of geoR [61] to explore and describe the horizontal spatial correlation patterns of the brightness temperature observation data by fitting the semivariogram model automatically at each assimilation step. The maximum distance considered when fitting the semivariogram of observation data was set at 10 km. Pairs of locations with separation distances larger than this value were ignored.

#### 4.2. Local Analysis of Brightness Temperature

In this assimilation experiment, we wanted to validate the combination of the horizontal spatial correlation and the OL analysis scheme of the LETKF, in which the separate analyses were performed for each model grid cell and the observations located in the local region surrounding the CLM grid cell were to be analyzed were selected to be assimilated. For the model grid cells covered by the synthetic observation, only one nearest local observation was assimilated. As for the comparison between the proposed local analysis scheme and the traditional method, we carried out six kinds of data assimilation scenarios, as follows: (1) Only 1 closest observation was used for each non-covered grid cell (1_Obs); (2) No more than 5 observations were used for each non-covered grid cell (5_Obs), the next closest 4 observations were also assimilated in addition to the closest observation; (3) No more than 9 observations were used for each non-covered grid cell (9_Obs), 8 additional observations were assimilated in the data assimilation procedure; (4) No more than 16 observations were used for each non-covered grid cell (16_Obs), with 15 additional observations assimilated in the data assimilation procedure; (5) No more than 25 observations were used for each non-covered grid cell (25_Obs), with 24 additional observations assimilated in the data assimilation procedure; (6) No more than 36 observations were used for each non-covered grid cell (36_Obs), with 35 additional observations assimilated in the data assimilation procedure.

During the assimilation step, the soil moisture and soil temperature were entered into CMEM to map the brightness temperature, which would be used in LETKF and compared with the synthetic observation data. Thus, the weighted soil moisture in the model state vector of LETKF for each grid cell after the *H* operator operation will be replaced by the brightness temperature instead. The dimension of the observation for each grid cell of different scenarios are as follows: no more than 1 for 1_Obs, no more than 5 for 5_Obs, no more than 9 for 9_Obs, no more than 16 for 16_Obs, no more than 25 for 25_Obs and no more than 36 for 36_Obs. Firstly we calculated the correlogram values of all observations for each model grid cell. Then, 1, 5, 9, 16, 25, or 36 local brightness temperature observations were chosen and the chosen observations with correlogram values greater than 0.1 were assimilated for each model grid cell. After the selection of local correlated observations, the observation error variances were divided by the correlogram value to do the OL.

## Results

The assimilation results were evaluated using the root mean square error (RMSE) values which were calculated according to:

$$\mathrm{RMSE}=\sqrt{\frac{\sum_{n=1}^{N}{\left(\text{Estimated}-\text{Truth}\right)}^{2}}{N}}$$ (14)

where “*Estimated*” is the ensemble mean of soil moisture without assimilation or the ensemble mean of soil moisture after assimilation, and *N* is the number of model time steps, which is 2184 for this study. The smaller the RMSE value is, the better the assimilation results will be.

The RMSE value of each grid cell in the whole assimilation time series was calculated firstly. Then the mean RMSE values for all covered and all uncovered grid cells were calculated separately using the RMSE values calculated before. Finally, the mean RMSE value for all 4340 grid cells (covered and uncovered) was calculated. We calculated the 95% confidence interval of the mean RMSE value using a Bayesian methodology, in which a non-informative prior was derived for the mean and variance and the Bayes rule was used to compute the posterior probability density function of mean and variance [62]. This function is implemented in the scientific python packages SciPy (scipy.stats.bayes_mvs – SciPy website. Available: http://www.scipy.org/. Accessed 2014 Dec 11).

**Figure 3:**
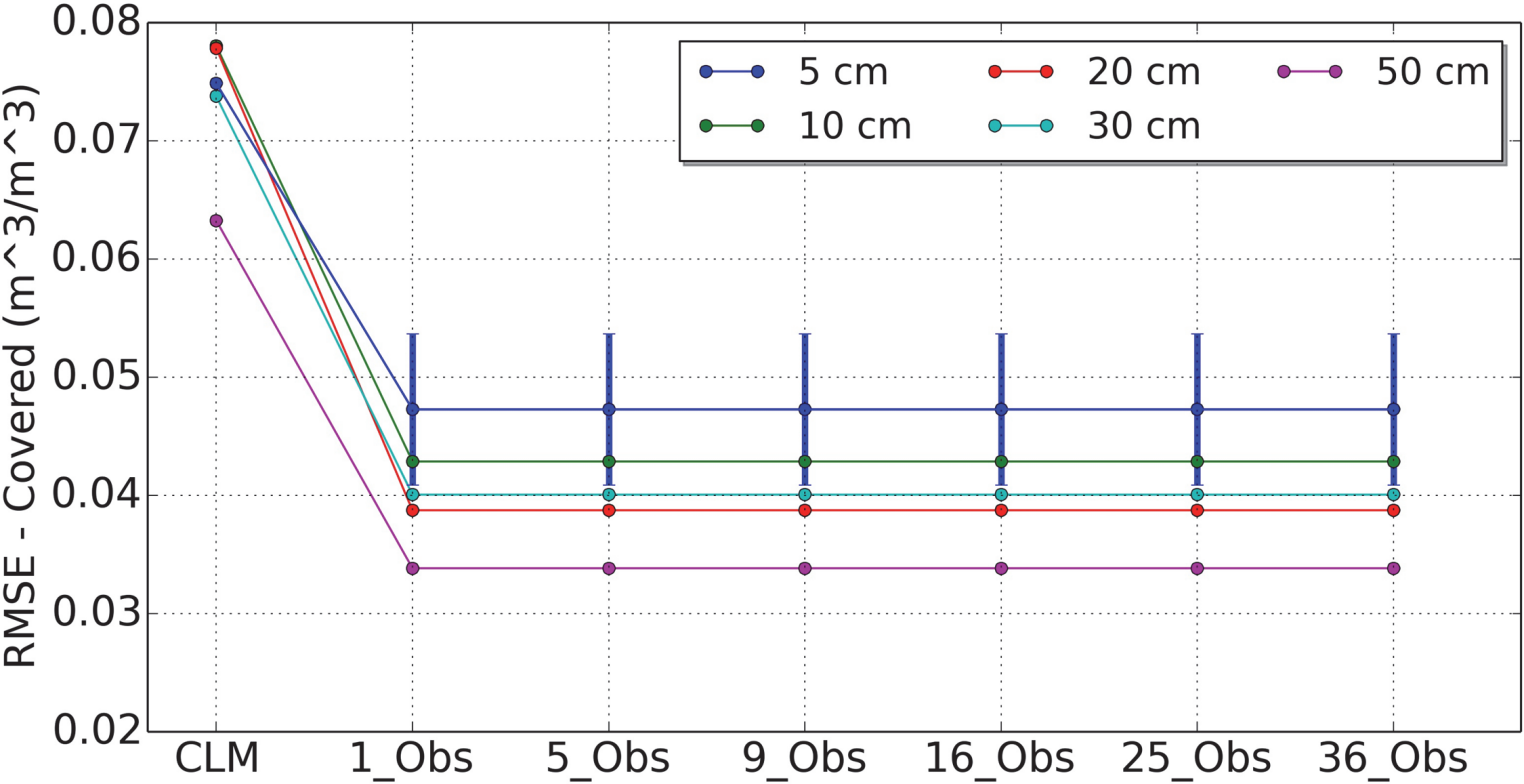
Mean RMSE values of covered area soil moisture for open loop run (CLM) and 6 assimilation strategies of 1_Obs, 5_Obs, 9_Obs, 16_Obs, 25_Obs and 36_Obs for depths of 5 cm, 10 cm, 20 cm, 30 cm, and 50 cm.

Fig. 3 shows the mean RMSE values soil moisture at the covered area by observation (including 95% confidence intervals) for open loop run and the other six assimilation scenarios of 1_Obs, 5_Obs, 9_Obs, 16_Obs, 25_Obs and 36_Obs for depths of 5 cm, 10 cm, 20 cm, 30 cm and 50 cm, respectively. It is clear that the soil moisture profiles of the covered grid area was improved for all scenarios compared with the open loop run (CLM). Because only one local observation was assimilated for each grid cell at the covered area, the results of different scenarios were same. The RMSE values were reduced by 35.9%, 37.9%, 41.1%, 42.9%, and 47.1% for depths of 5 cm, 10 cm, 20 cm, 30 cm and 50 cm, respectively.

**Figure 4:**
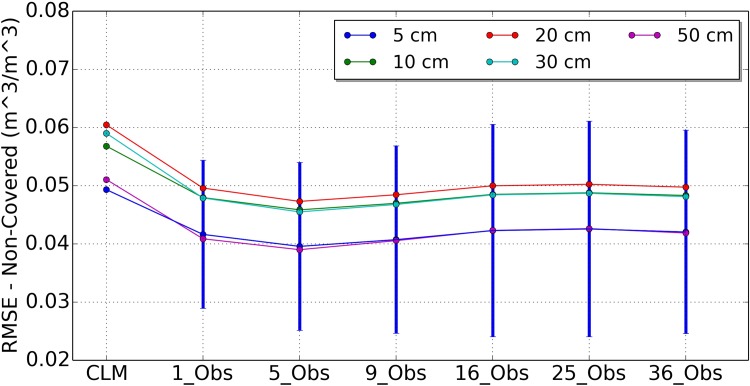
Mean RMSE values of uncovered area soil moisture for open loop run (CLM) and 6 assimilation strategies of 1_Obs, 5_Obs, 9_Obs, 16_Obs, 25_Obs and 36_Obs for depths of 5 cm, 10 cm, 20 cm, 30 cm, and 50 cm.

Fig. 4 shows the RMSE values of soil moisture where the observations did not cover. The results differ from those of Fig. 3. If five or nine local observations were assimilated, the results of uncovered grid cells were improved obviously. The reductions of RMSE value for scenario 1_Obs are 31.8%, 33.0%, 36.7%, 41.4% and 46.2% for depths of 5 cm, 10 cm, 20 cm, 30 cm and 50 cm, respectively. The reductions of RMSE value for scenario 5_Obs are 35.0%, 36.2%, 39.3%, 42.9% and 47.6% for depths of 5 cm, 10 cm, 20 cm, 30 cm and 50 cm, respectively. The reductions of RMSE value for scenario 9_Obs are 35.2%, 36.3%, 39.3%, 42.7% and 46.7% for depths of 5 cm, 10 cm, 20 cm, 30 cm and 50 cm, respectively. The results of scenario 5_Obs were quite similar to the results of scenario 9_Obs. The results of scenario 9_Obs in upper layers are better than that of scenario 5_Obs. If more than 9 local observations were assimilated, the results become worse than less local observations.

**Figure 5:**
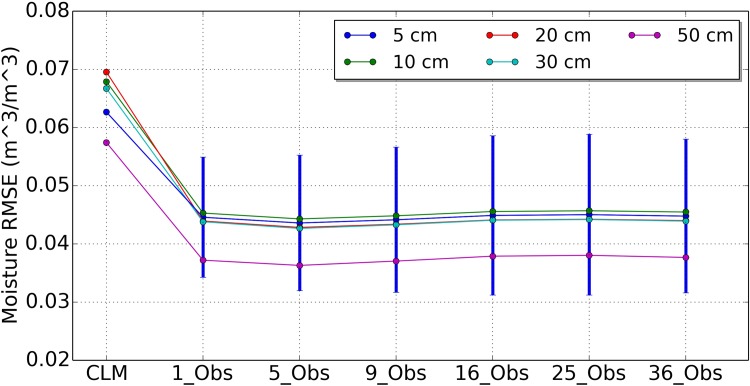
Mean RMSE values of whole catchment soil moisture for open loop run (CLM) and 6 assimilation strategies of 1_Obs, 5_Obs, 9_Obs, 16_Obs, 25_Obs and 36_Obs for depths of 5 cm, 10 cm, 20 cm, 30 cm, and 50 cm.

Fig. 5 plots the soil moisture mean RMSE values for open loop run (CLM) and the other six assimilation scenarios of 1_Obs, 5_Obs, 9_Obs, 16_Obs, 25_Obs and 36_Obs for depths of 5 cm, 10 cm, 20 cm, 30 cm and 50 cm at basin scale. From these results, we can see that the overall impacts of different numbers of local brightness temperature observations used in OL on the soil moisture estimation for the whole catchment. Like the separate results in Fig. 4, both scenario 5_Obs and scenario 9_Obs improved the soil moisture estimation similarly. These results are better than that of the scenario 1_Obs. The RMSE values of scenario 9_Obs were reduced by 35.6%, 37.2%, 40.2%, 42.8%, 39% and 46.9% for depths of 5 cm, 10 cm, 20 cm, 30 cm and 50 cm, respectively.

We can see from Fig. 5 that the OL analysis was further improved when more local observations were involved in assimilation for the uncovered model grid cells, but with an upper bound of 9 local observations. These results demonstrated the positive impacts of the horizontal correlated observations on the uncovered grid cells. Moreover, the soil moisture profile at the grid cells without observations could be improved using the OL analysis scheme. The standard deviation of the RMSE values for the 5 cm depth of difference scenarios are plotted in Fig. 3, Fig. 4 and Fig. 5. The error bar of 16_Obs, 25_Obs and 36_Obs became larger than 1_Obs, 5_Obs and 9_Obs. This results are consistent with the average RMSE values.

**Figure 6:**
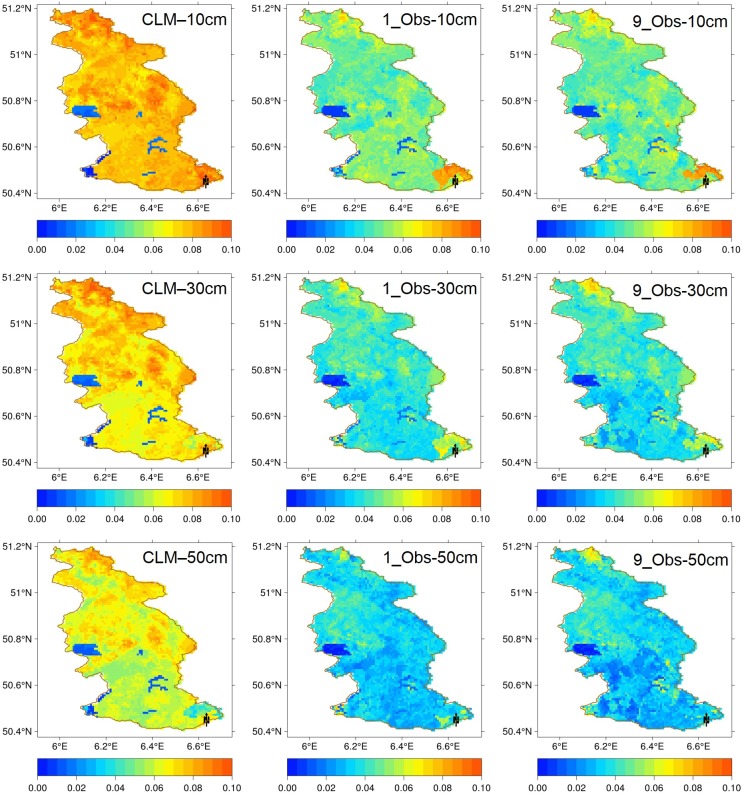
The basin scale soil moisture RMSE values for reference run (Truth), open loop run (CLM), and the assimilation strategy of 9_Obs at depths of 10 cm, 30 cm, and 50 cm.

Fig. 6 shows the soil moisture RMSE values for reference run, open loop run, and the scenario of 9_Obs at the basin scale. The reductions of RMSE values are very clear at the non-forest area and forest area when one local observation is used in the assimilation of each model grid cell. If 9 local observations were used, the RMSE values decrease further from the last column of Fig. 6.

**Figure 7:**
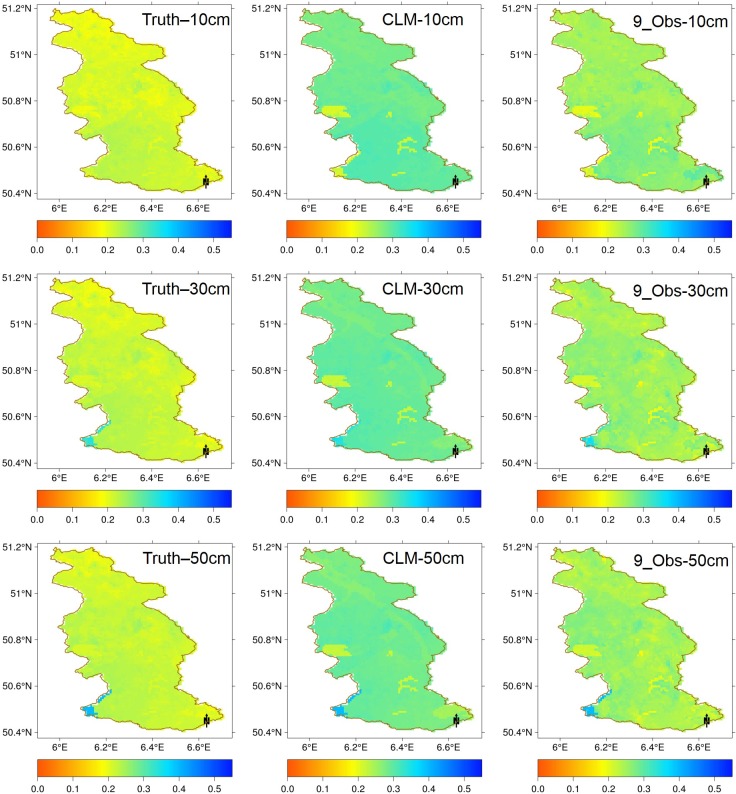
The basin scale average soil moisture for reference run (Truth), open loop run (CLM), and the assimilation strategy of 9_Obs at depths of 10 cm, 30 cm, and 50 cm.

Fig. 7 plots the averaged soil moisture of three months for reference run, open loop run, and the scenario of 9_Obs at the basin scale. These figures show the improvements of the soil moisture spatially. It is obvious that the soil moisture results for depths of 10 cm, 30 cm and 50 cm of scenario 9_Obs are closer to the reference run (Truth) than the open loop run (CLM). In order to compare the soil moisture pattern quantitatively, the Hausdorff distance (HD) between the open loop run and the reference run, and between the scenario 9_Obs and the reference run were calculated, respectively. The HD measures the spatial similarity of points in two finite sets [63]. Lower HD values mean high similarity. The HD values of soil moisture for open loop run at 10 cm, 30 cm and 50 cm are 0.66, 0.60, and 0.55, respectively. The HD values of soil moisture for scenario 9_Obs at 10 cm, 30 cm and 50 cm are 0.47, 0.31, and 0.28, respectively. Based on these HD values, we can see that the soil moisture spatial pattern of 9_Obs bocame closer to the reference than the open loop run.

## Discussion

Due to the spatial availability of the measurements, it is very common that all model grid cells cannot be measured at the same observation time. Thus, the question of how to do data assimilation for the model grid cells without observations has been proposed, and studies have paid more attention to the spatial horizontal transfer of observations in data assimilation. The studies proved that the model states where the observations were not available could be updated using the local correlated observations. In this study: (1) the geostatistical semivariogram model was used to fit the microwave brightness temperature data and analyze the spatial correlation characteristics of observation. This is different from the commonly used subjective correlation function and constant spatial correlation range; (2) the derived spatial correlation model and correlation range at each assimilation step were combined with LETKF and OL to update the model grid cells without observations.

The local correlated observations needed to be chosen for each model grid cell before the data assimilation in OL and LETKF. However, this step is very subjective in the previous studies. Moreover, the subjective distance-dependent function is also needed in the OL. We tried to use the geostatistical analysis to obtain a more reasonable representation of this subjective distance-dependent function. This function determines which observations can be used in the analysis for each model grid cell based on the spatial correlation of observation data. The objective of spatial correlation analysis is to choose the relevant observations for each grid cell, not to model the spatial error correlation. Generally, we assigned a uniform observation error for simplicity because of the limited information on observation error.

For the model grid cells covered by the observation we only used one local observation in the assimilation, because we found that more local observations could deteriorate the assimilation results for the covered grid cell. However, for the grid cells not covered by the observation, the above results show that more local observations involved in the assimilation could be useful, but with the upper bound of 9 local observations. In data assimilation it is not necessary to have observations for each grid cell. However, we need to know how to use the neighboring observations to update the uncovered grid cells and how many observations could be trusted and assimilated for each model grid cell. If we have a lower number of observations (compared with the number of model grid cells), the observations located at different local surrounded grid cells could then be used for the assimilation of each model grid cell because of the spatial horizontal correlation contained in the background error covariance.

The results of spatial correlation analysis also depend on the geostatistical semivariogram fitting methods used in the study. In this study, the methods provided by the R package geoR were used to fit the geostatistical semivariogram. Moreover, the spatial correlation imposed on the atmospheric forcing data could also influence the localization results. The semivariogram models used in our experiments were assumed to be isotropic for simplicity. It is an ideal situation in the horizontal spatial correlation statistics. An anisotropic model, however, may be more reasonable because of the intrinsic spatial heterogeneity contained in the soil moisture distribution. Moreover, the horizontal spatial correlation structures of the soil moisture and remote sensing observations are different in different observation scales. Thus, the horizontal spatial correlation pattern can be thoroughly exploited.

Usually the constant localization lengths were used in the LETKF. Many works have studied the sensitivity of analysis error to the localization scale in LETKF and have shown that there is an optimal localization length in LETKF [31,33,34], and showed that the optimal localization length is dependent on the ensemble size and observing network [33]. If the shorter or larger localization length were to be used in LETKF, the results would become worse. Larger localization length also means that more observations would be used in the assimilation, so more observations would increase the analysis errors. In our study, we used the different localization length which was estimated at each assimilation step. The increased number of observations is similar to the way of increasing the localization length. In the background error covariance localization (CL), which was used in ETKF, there was also an optimal localization length [31]. Beyond this length scale, the analysis error will be increased. The increased analysis error is due to the spurious covariance when more observations were involved. With larger localization length, the system could be dominated by the spurious observation increments that prevent it from converging to the truth [31,34].

Generally, the land data assimilation is assumed to be used to remove the white noise of land surface model, but the model bias sometimes cannot be removed because of the biased input. In this study, we assumed that the soil sand fraction, soil clay fraction, and organic matter density were biased, and the biased soil moisture mainly due to the soil parameters. The assumption was we also could not get the high quality of sand, clay, and organic data for each model grid cell in reality. It was difficult to make the model simulation with white noise only because of the biased soil parameters. Moreover, we could not obtain enough measurement data to calibrate the distributed land surface model at the catchment scale. For example, when the land surface model is used in irrigated farmland, the simulated soil moisture will be biased without the irrigation as input data. However, the benefit of land data assimilation is to remove this bias with observation.

Only two layers of soil properties could be obtained from the global soil database. There is no general rule to interpolate these two layers’ soil data into 10 layers. In this study, the linear interpolation was used to prepare 10 layers of CLM soil properties. This would make the soil moisture of difference layers highly correlated. The soil moisture of the deep layers can be easily updated based on the correlation between the deep layer and surface layer. Therefore, the synthetic experiment could overestimate the performance of assimilation.The applicability of the proposed local analysis scheme is only validated at the microscale catchment where the soil moisture horizontal spatial correlation can be relevant and the local analysis can be clearly beneficial. The possible extension of this methodology to the mesoscale assimilation of the satellite microwave observation remains to be verified. The results indicate a preliminary framework for combining the LETKF with the geostatistical horizontal spatial correlation representation. The spatial resolution of the L-band microwave brightness temperature data used in this study is higher than the common available microwave sensors, such as SMOS mission [5] and SMAP mission [4]. Many ongoing studies are trying to downscale the coarse microwave brightness temperature data [64] or soil moisture product for the catchment scale application [65,66,67,68]. To assimilate the coarse microwave brightness temperature data at catchment scale, a prior downscaling is needed. If the spatial patter of soil moisture is controlled by the precipitation mainly, and the land surface model (or hydrological model) can simulate the spatial pattern of soil moisture well, then the prior downscaling of coarse soil moisture is not necessary, in which the data assimilation can be used to downscale and assimilate the coarse soil moisture product successfully [69]. For many cases, however, the spatial pattern of soil moisture is influenced by the irrigation, and moreover the large-scale irrigation data are not available for the modeling generally. Then the model cannot catch the spatial pattern of soil moisture, and the coarse soil moisture also cannot catch the spatial pattern of fine scale soil moisture. For this case, we need to use more prior knowledge (such as the Normalized Difference Vegetation Index—NDVI, Temperature Vegetation Dryness Index—TVDI) to downscale the coarse soil moisture [67] or brightness temperature [64] before assimilation. In this study, we assumed that the high-resolution downscaled microwave brightness temperature data were available for data assimilation application.

## Conclusions

The synthetic brightness temperature assimilation was carried out at catchment scale with the land surface model CLM, and considered the spatial availability of the L-band microwave remote sensing under forest area. The perturbed atmospheric forcing and soil parameter were used to describe the model uncertainties. The horizontal spatial correlation characterizations of microwave brightness temperature data were fitted using the geostatistical semivariogram and incorporated into LETKF analysis to solve the problem of spatial availability of observations by means of OL. The selection of local correlated brightness temperature observations in OL considered the observations located in a local region surrounding the model grid cell to be assimilated, and depended on the observational spatial correlated characteristics, which was modeled using the geostatistical semivariogram fitting methods.

Six separate assimilation scenarios were carried out to evaluate the performance of the combination of the OL and the geostatistical spatial correlation representation in soil moisture estimation. The first scenario was 1_Obs, in which the model grid cells were updated with no more than 1 nearest observation; the second was the scenario 5_Obs, in which the model grid cells were updated with no more than 5 local observations; the third was the scenario 9_Obs, in which the model grid cells were updated with no more than 9 local observations; similarly no more than 16, 25, or 36 local observations were evaluated in different assimilation scenarios of 16_Obs, 25_Obs, and 36_Obs. We incorporated the local observation selection and OL to update all the model grid cells with available correlated observations surrounding the analysis grid cells in all cases. From the results we can conclude that more local brightness temperature data assimilated will improve the estimation of model grid cells without observation data, but with an upper bound of 9 local observations in this case. The use of local analysis in the microwave brightness temperature data assimilation has proven to correct the model errors in the soil moisture profile at the catchment scale.

This study demonstrates the potentials of OL and geostatistical spatial correlation representation to improve the soil moisture analysis using the L-band microwave brightness temperature data, which cannot cover the study area fully in space due to the effects of forest. The geostatistical distance-dependent functions have been adopted to analyze the horizontal spatial correlation characteristics of the L-band microwave brightness temperature data. The finding of 9 local observations is case dependent. The optimal number of local observations needs to be investigated for the specific application, because the spatial correlation characteristics will change along the changing environmental conditions. Moreover the model subsurface physics of water vertical movement also influences the results of deep layers assimilation [59].

This scheme will also be helpful in minimizing the errors in the spatial registration that will result in spatial mismatch of remote sensing images. Because of the spatial mismatch of remote sensing data, it will be useful to find many local surrounded observations instead of the exact nearest observation to be used in data assimilation. Another approach to handle the spatial availability of observations is to do the spatial interpolation before data assimilation, such as the kriging method [36]. However, there are also spatial registration errors in the remote sensing products [70]. The results of these errors will make it difficult to find the exact nearest observation for each model grid cell. Thus, the OL approach provides us the opportunity to bypass the spatial registration errors.
